# Dual Loading Antiplatelet Therapy in Patients With Acute Coronary Syndrome and High Bleeding Risk Undergoing Percutaneous Coronary Intervention: Findings From the Improving Care for Cardiovascular Disease in China Project

**DOI:** 10.3389/fcvm.2022.774123

**Published:** 2022-03-23

**Authors:** Yan Yan, Wei Gong, Xin Huang, Siyi Li, Ge Wang, Youcai Ma, Yongchen Hao, Jun Liu, Shaoping Nie

**Affiliations:** ^1^Department of Cardiology, Center for Coronary Artery Disease, Beijing Anzhen Hospital, Capital Medical University, Beijing, China; ^2^National Clinical Research Center for Cardiovascular Diseases, Beijing, China; ^3^Department of Cardiology, Beijing Shijitan Hospital, Capital Medical University, Beijing, China; ^4^Department of Epidemiology, Beijing Anzhen Hospital, Beijing Institute of Heart, Lung and Blood Vessel Diseases, Capital Medical University, Beijing, China

**Keywords:** acute coronary syndrome, high bleeding risk, dual antiplatelet loading, in-hospital outcome, CCC-ACS registry

## Abstract

**Objective:**

Loading dose of dual antiplatelet therapy (LD) is supported by the guidelines for patients with acute coronary syndrome (ACS). However, limited data is provided in the series of high bleeding risk (HBR) patients with ACS and percutaneous coronary intervention (PCI).

**Methods:**

Using data from the Improving Care for Cardiovascular Disease in China—Acute Coronary Syndrome registry, conducted between 2014 and 2019, we stratified all ACS patients with HBR and PCI according to LD used within 24 h of first medical contact or not. Inverse probability of treatment weighting (IPTW) and Cox proportional hazards model with hospital as random effect were used to analyze differences in in-hospital clinical outcomes: the primary efficacy endpoint was mortality, and the primary safety endpoint was bleeding.

**Results:**

Of 21,654 evaluable patients 14,322 (66.2%) were treated with LD, and were on average older, less likely to have comorbidities and higher hemoglobin, more often treated GPI and anticoagulant during hospitalization than those without LD. After IPTW adjustment for baseline differences, LD was associated with significantly increased risk of in-hospital mortality [1.89 vs. 1.02%; hazard ratio (HR): 1.71 (95% confidence interval 1.12, 2.42); *p* < 0.001] and in-hospital bleeding [3.89 vs. 3.3%; HR: 1.25 (1.03, 1.53); *p* = 0.03].

**Conclusions:**

In ACS patients with HBR, LD was associated with an increased risk of in-hospital mortality and bleeding complications after PCI. Dedicated randomized trials with contemporary ACS management are needed to confirm these findings.

## Key Questions

### What Is Already Known About This Subject?

There is currently a lack of data among the high-bleeding risk (HBR) population on the use of dual antiplatelet loading (LD) with a diagnosis of ACS and undergone PCI, with concerns that the potential bleeding risk may outweigh any benefit in terms of cardiovascular event reduction.

### What Does This Study Add?

Of 21,654 evaluable HBR—ACS patients who underwent PCI and were included in the CCC-ACS registry in China (2014–2019), 66.2% were treated with LD.

After adjustment for baseline differences, LD was associated with a significantly increased risk of in-hospital mortality [1.89 vs. 1.02%; hazard ratio (HR): 1.71 (95% confidence interval 1.12, 2.42); *p* < 0.001] and in-hospital bleeding [3.89 vs. 3.3%; HR: 1.25 (1.03, 1.53); *p* = 0.03].

### How Might This Impact on Clinical Practice?

This analysis of data—excluding HBR patients who received antiplatelet therapy without getting with the guidelines or with a chronic use of antiplatelet therapy—provides evidence that LD may indeed increase in-hospital mortality and bleeding after PCI for HBR-ACS, after adjustment for key baseline characteristics.

The results of this analysis provide the basis for further investigation on the use of LD in HBR-ACS patients, calling for bleeding risk stratification ahead of LD decisions in daily practice.

## Introduction

Dual antiplatelet therapy, including aspirin and a P2Y_12_ inhibitor, has become the cornerstone for treating patients with acute coronary syndrome (ACS) undergoing percutaneous coronary intervention (PCI). Administration of a loading dose of dual antiplatelet therapy (DAPT) is supported by the American College of Cardiology (ACC)/American Heart Association (AHA) and European Society of Cardiology (ECS) guideline (1, 2). However, recommendations in the guidelines are the same for all patients, neglecting their bleeding risks.

Recently, the Academic Research Consortium for High Bleeding Risk (ARC-HBR) has been proposed to standardize the definition of High Bleeding Risk (HBR), which was arbitrarily defined as a Bleeding Academic Research Consortium (BARC) 3 or 5 bleeding ≥ 4% at 1-year (3). According to this criteria, a reporting from the all-comers registry in Japan, 43% of patient represented of HBR and with 3-fold risk of bleeding event than the non-HBR patients (4). Furthermore, prolonged duration of antiplatelet therapy, which mean to mitigate ischemic events, lead to an inordinate increase in bleeding events in long-term observation of HBR population (5). However, as a common usage of antiplatelet therapy, dual loading dose (LD) in HBR patients with ACS, was with limited data regarding the in-hospital outcomes. In this study, we aimed to evaluate whether receiving a dual loading dose of antiplatelet agents is appropriate for HBR patients with ACS and PCI during hospitalization.

## Methods

### Study Design

We used data from the nationwide, real-world registry of CCC-ACS (Improving Care for Cardiovascular Disease in China). Launched in 2014, the CCC-ACS project (clinicaltrial.gov NCT 02306616) is a prospective registry including 159 tertiary and 82 secondary hospitals across China. The registry is a common program of the AHA and the Chinese Society of Cardiology (CSC), recruiting patients with ST-segment elevation myocardial infarction (STEMI) and non-ST elevation acute coronary syndromes (NSTE-ACS). Detailed information has been reported previously (6). Briefly, eligible cases in each participated site are reported by trained data abstractors every month, while the acute myocardial infarction cases have reporting priority.

Baseline characteristics, procedural data, in-hospital treatment and outcomes are recorded via an electronic data capture platform by trained data abstractors. Standardized definitions are utilized across all hospitals for variable collection (7). Third-party clinical research associates were in charge of quality assurance. Audit reports were fed back to each center regularly for quality control. The study was approved by the institutional review board at Beijing Anzhen Hospital, Capital Medical University.

### Study Population

As of 31th December 2019, on the basis of principal discharge diagnosis, 113,650 patients with ACS have been registered. Of these, 21,654 patients with HBR who received a known dose of both aspirin and P2Y_12_ inhibitor within 24 h of first medical contact and received PCI were included in this study. Patients who received antiplatelet therapy without getting with the guideline recommendations (e.g., only aspirin loading, only P2Y_12_ loading or loading with both ticagrelor and clopidogrel), received aspirin or P2Y_12_ inhibitor within 2 weeks before admission were excluded from the analysis. Patients admitted at a situation that could not be treated with oral dual loading (e.g., active bleeding, out of hospital cardiac arrest) were also excluded.

### Variable Definitions

On the basis of data availability, some of the ARC-HBR criteria were not available or need to be modified. The comparison of definitions between ARC-HBR criteria and the currents study were listed in the Supplementary Table 1. ARC-HBR criteria applied in this study are as follows: Age ≥ 75 years (minor), eGFR 30–59 mL/min (minor); Hemoglobin 11–12.9 g/dL for men and 11–11.9 g/dL for women (minor); bleeding tendencies before admission (minor); long-term use of aspirin before admission (minor); prior ischemic stroke or TIA (minor); Warfarin usage before admission (major); eGFR < 30 mL/min (major); Hemoglobin < 11 g/dL (major); platelet count < 100 × 10^9^/L (major); prior hemorrhagic or ischemic stroke (major); medical history of surgery or tooth extraction (major). Patients were classified to HBR patients if at least one major or two minor ARC-HBR criteria were met.

Patients were considered as LD if they received dual loading with both aspirin and P2Y_12_ inhibitor (clopidogrel or ticagrelor) within 24 h of first medical contact. The administration of aspirin and clopidogrel or ticagrelor with a dose of over 100 and 300 mg or 180 mg, respectively, was defined as LD. Patients who received loading with neither aspirin nor P2Y_12_ inhibitor were defined as No LD. The information of the type and dose of antiplatelet therapy received within 24 h of first medical contact were specifically entered into the registry.

### Endpoints

The primary efficacy endpoint was in-hospital mortality. The primary safety endpoint was in-hospital bleeding. In detail, stent thrombosis was defined as an acute/subacute stent occlusion after procedure. The event “myocardial infarction (MI)” corresponds in our study to a “reinfarction” within the hospitalization following the index MI. The stroke was defined as a new neurological deficit during hospitalization. Major bleeding was defined as any of the following events: fatal bleeding, intracranial bleeding, retroperitoneal bleeding, and drop in hemoglobin ≥ 40 g/L during hospitalization, transfusion with overt bleeding, or bleeding requiring surgical intervention. Fatal bleeding was defined as any death within 7 days following a major bleeding. All of these endpoints were reported by clinical doctors and documented in medical records during hospitalization.

### Statistical Analysis

Baseline characteristics and procedural details of ACS patients were described. Continuous variables are presented as a mean and standard deviation (SD) and categorical variables are presented as frequency and percentage. *T*-test for continuous variables and Chi-square test for categorical variables were used to test the statistical differences between groups. Kaplan-Meier methods were used to estimate 30-days event rates for each endpoint, and comparisons between study groups were performed using log-rank test.

To consolidate the findings, we also carried out inverse probability of treatment weighting (IPTW) using the propensity score method in study cohort and compared difference among two groups. A logistic regression was used to estimate propensity score, adjusting following variables: age, gender, previous disease history (MI, PCI, CABG, hypertension, dyslipidemia, diabetes, renal failure history, heart failure history, peripheral artery disease, ischemic stroke, hemorrhagic stroke), current smoker, cardiogenic shock, Killip class, systolic blood pressure, heart rate, elevated troponin (Tn)T or TnI, hemoglobin, diagnosis of STEMI, Global Registry of Acute Coronary Events (GRACE) score, preadmission of aspirin and P2Y_12_ inhibitor, DAPT at arrival, glycoprotein IIb/IIIa inhibitors (GPI) at arrival, ACEIs/ARBs or β-blockers at admission, PCI access and drug-eluting stent. IPTW was calculated by each individual base on his/her propensity score (*PS*). Each case of LD group was given a weight of *Pt/PS*, and each case from the no LD group was given a weight *(1-Pt)/(1-PS)*, where *Pt* refers to the percentage of patients receiving any LD among the whole cohort. By this way we could get a stabilized weight for each case of the study cohort, avoiding any extreme values that may result in unreliable outcomes.

Cox proportional hazard models were used to analyze the effects of LD on each endpoint, and Hazard ratios (HR) and 95% confidence intervals (95% CI) were reported. Two models were applied to explore the association between LD and endpoints: (1) an unadjusted model; and (2) an adjusted model controlling for prespecified variables with impact of in-hospital outcomes (gender, medical history, current smoker, cardiogenic shock, Killip class, elevated TnT or TnI, anticoagulant during hospitalization, year of enrolment, and hospital type). Subgroup analysis among thirteen subsets was performed to assess the consistency of treatment effects on primary efficacy endpoint and safety endpoint.

Imputation was performed for variables with missing data with the sequential regression multiple imputation method by IVEware software version 0.2 (Survey Research Center, University of Michigan, Ann Arbor, MI, USA). No LD was used as reference in all analyses. All tests were two-sided, and a *P*-value of <0.05 was considered to indicate statistical significance. All analyses were performed using SAS version 9.3 (SAS Institute Inc.; Cary, NC) statistical software.

### Patient and Public Involvement

This research was done without patient involvement.

## Results

### Patient's Characteristics

**Figure 1:**
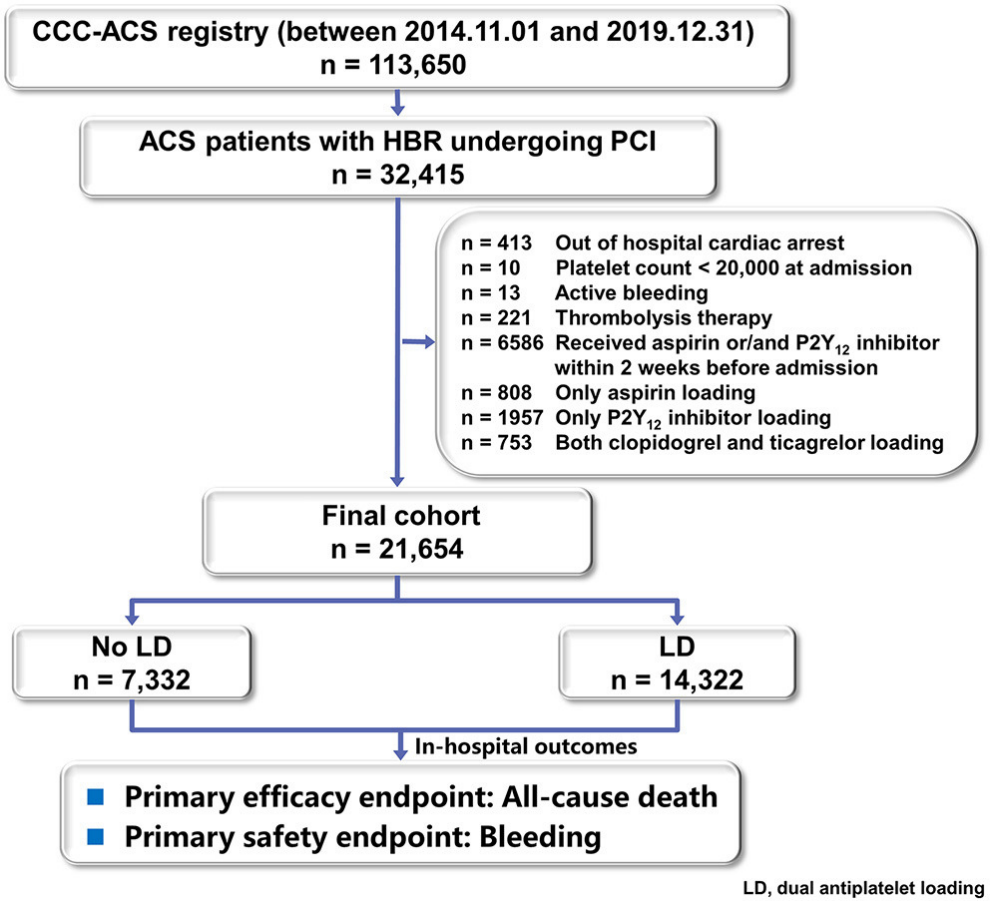
Study flow chart.

Of 113,650 diagnosed ACS patients enrolled into the CCC-ACS database between November 2014 and December 2019, 21,654 patients with HBR and PCI were analyzed as the final population (Figure 1). Of them, 7,332 were classified in the no LD group and 14,322 in the LD group. Baseline characteristics and treatment of the study population were presented in Table 1. Patients receiving a LD were on average older, less likely to have heart failure history and prior ischemic stroke. They presented with worse cardiac function and higher hemoglobin level. They were less likely to receive in-hospital ACEIs/ARBs or β-blockers. But they were prescribed more frequently of GPI and anticoagulant during hospitalization. Among LD patients, PCI was more frequently performed through radical access with drug-eluting stent. The use of potent P2Y_12_ inhibitor, ticagrelor, was less frequent at 40.7% in the LD patients. After adjusting with IPTW method, baseline characteristics were well balanced (Table 1).

**Table 1:** Baseline characteristics.

**Characteristics**	**Unweighted population**			**Inverse probability of treatment weighting (IPTW) population**		
	**LD (*n* = 14,322)**	**No LD (*n* = 7,332)**	***P*** **-value**	**LD (*n* = 14,322)**	**No LD (*n* = 7,332)**	***P*** **-value**
**Socio-demographic**						
Age, mean ± SD, years	68.6 ± 11.6	68.1 ± 11.2	<0.001	68.4 ± 11.4	68.5 ± 11.2	0.59
Age ≥ 65 years	9,222 (64.4)	4,756 (64.9)	0.49	9,222 (64.5)	4,756 (64.8)	0.61
Female	4,514 (31.5)	2,266 (30.9)	0.36	4,514 (31.1)	2,266 (31.2)	0.91
**Medical history and risk factors**						
Previous MI	507 (3.5)	283 (3.9)	0.23	507 (3.6)	283 (3.7)	0.85
Previous PCI	547 (3.8)	300 (4.1)	0.33	547 (3.9)	300 (4.0)	0.57
Previous CABG	26 (0.2)	12 (0.2)	0.77	26 (0.2)	12 (0.2)	0.79
Hypertension	8,155 (56.9)	4,223 (57.6)	0.36	8,155 (57.3)	4,223 (57.3)	0.98
Dyslipidemia	775 (5.4)	443 (6.0)	0.06	776 (5.6)	443 (5.6)	0.89
Diabetes mellitus	3,389 (23.7)	1,781 (24.3)	0.31	3,389 (23.9)	1,781 (23.9)	0.95
Renal failure history	272 (1.9)	145 (2.0)	0.69	272 (1.9)	145 (1.9)	0.94
Heart failure history	114 (0.8)	80 (1.1)	0.03	114 (0.9)	80 (0.9)	0.78
Peripheral artery disease	120 (0.8)	51 (0.7)	0.26	120 (0.8)	51 (0.8)	0.97
Hemorrhagic stroke	507 (3.5)	283 (3.9)	0.23	204 (1.5)	119 (1.5)	0.98
Ischemic stroke	1,885 (13.2)	1,157 (15.8)	<0.001	1,885 (13.9)	1,157 (13.9)	0.9
Current smoker	4,757 (33.2)	2,659 (36.3)	<0.001	4,757 (34.4)	2,659 (34.0)	0.5
**Clinical Status at admission**						
Heart rate, mean ± SD, bpm	77.2 ± 16.7	77 ± 15.9	0.43	77.1 ± 16.4	77.2 ± 16.1	0.8
SBP, mean ± SD, mmHg	129.4 ± 24.7	128.4 ± 23.6	0.01	129.0 ± 24.1	129.1 ± 24.0	0.82
Killip class						
I	10,018 (70.0)	4,589 (62.6)	<0.001	10,018 (67.7)	4,589 (67.0)	0.58
II or III	3,578 (25.0)	2,419 (33.0)	3,578 (27.6)	2,419 (28.2)	
IV	726 (5.1)	324 (4.4)	726 (4.8)	324 (4.9)	
Type of ACS						
STEMI	10,269 (71.7)	4,496 (61.3)	<0.001	10,269 (68.4)	4,496 (67.5)	0.17
NSTE-ACS	4,053 (28.3)	2,836 (38.7)		4,053 (31.6)	2,836 (32.5)	
GRACE score >140	525 (3.7)	301 (4.1)	0.11	525 (3.8)	301 (3.8)	0.93
**Laboratory examinations**						
Hemoglobin, mean ± SD, g/L	127.7 ± 20.5	123.9 ± 19.7	<0.001	126.6 ± 20.5	126.2 ± 19.5	0.19
Creatinine, mean ± SD, μmol/dL	98.1 ± 80.6	100.6 ± 81.3	0.03	98.3 ± 81.4	100.0 ± 77.7	0.13
**In-hospital medication**						
Aspirin loading	14,322 (100)	0 (0)	<0.001	14,322 (100)	0 (0)	<0.001
Ticagrelor loading	5,829 (40.7)	0 (0)	<0.001	5,829 (40.1)	0 (0)	<0.001
Clopidogrel loading	8,493 (59.3)	0 (0)	<0.001	8,493 (59.9)	0 (0)	<0.001
ACEIs/ARBs	6,592 (46.0)	3,643 (49.7)	<0.001	6,592 (47.8)	3,643 (47.3)	0.5
β-blockers	7,428 (51.9)	4,123 (56.2)	<0.001	7,428 (54.1)	4,123 (53.6)	0.46
Statins	13,705 (95.7)	6,806 (92.8)	<0.001	13,705 (95.8)	6,806 (94.9)	<0.001
GP IIb/IIIa inhibitor	4,987 (34.8)	2,157 (29.4)	<0.001	4,987 (33.5)	2,157 (33.0)	0.52
**Anticoagulant therapy during hospitalization**						
None	3,780 (26.4)	2,025 (27.6)	0.05	3,780 (26.6)	2,025 (26.0)	0.35
UFH	725 (5.1)	255 (3.5)	<0.001	725 (5.0)	255 (3.6)	<0.001
LMWH	9,734 (68.0)	4,867 (66.4)	0.02	9,734 (67.8)	4,867 (67.9)	0.9
Fondaparinux	192 (1.3)	163 (2.2)	<0.001	192 (1.3)	163 (2.3)	<0.001
Other	149 (1.0)	144 (2.0)	<0.001	149 (1.1)	144 (2.0)	<0.001
**Procedural details**						
Radial access	9,553 (66.7)	4,087 (55.7)	<0.001	9,543 (63.4)	4,087 (62.3)	0.12
Stent implantation	8,971 (62.7)	3,885 (53.0)	<0.001	8,971 (59.8)	3,885 (58.9)	0.19
Drug-eluting stent implanted	9,060 (63.3)	3,917 (53.4)	<0.001	9,060 (60.5)	3,917 (59.4)	0.15
In-admission CABG after PCI	70 (0.5)	55 (0.8)	0.02	70 (0.5)	55 (0.6)	0.22

*Values are numbers (percentages) unless otherwise indicated. Percentages may not sum to 100 due to rounding*.

*LD, dual antiplatelet loading; MI, myocardial infarction; PCI, percutaneous coronary intervention; CABG, coronary artery bypass grafting; SBP, systolic blood pressure; UFH, unfractionated heparin; LMWH, low-molecular-weight heparin*.

### Clinical Outcomes and Association With LD

**Figure 2:**
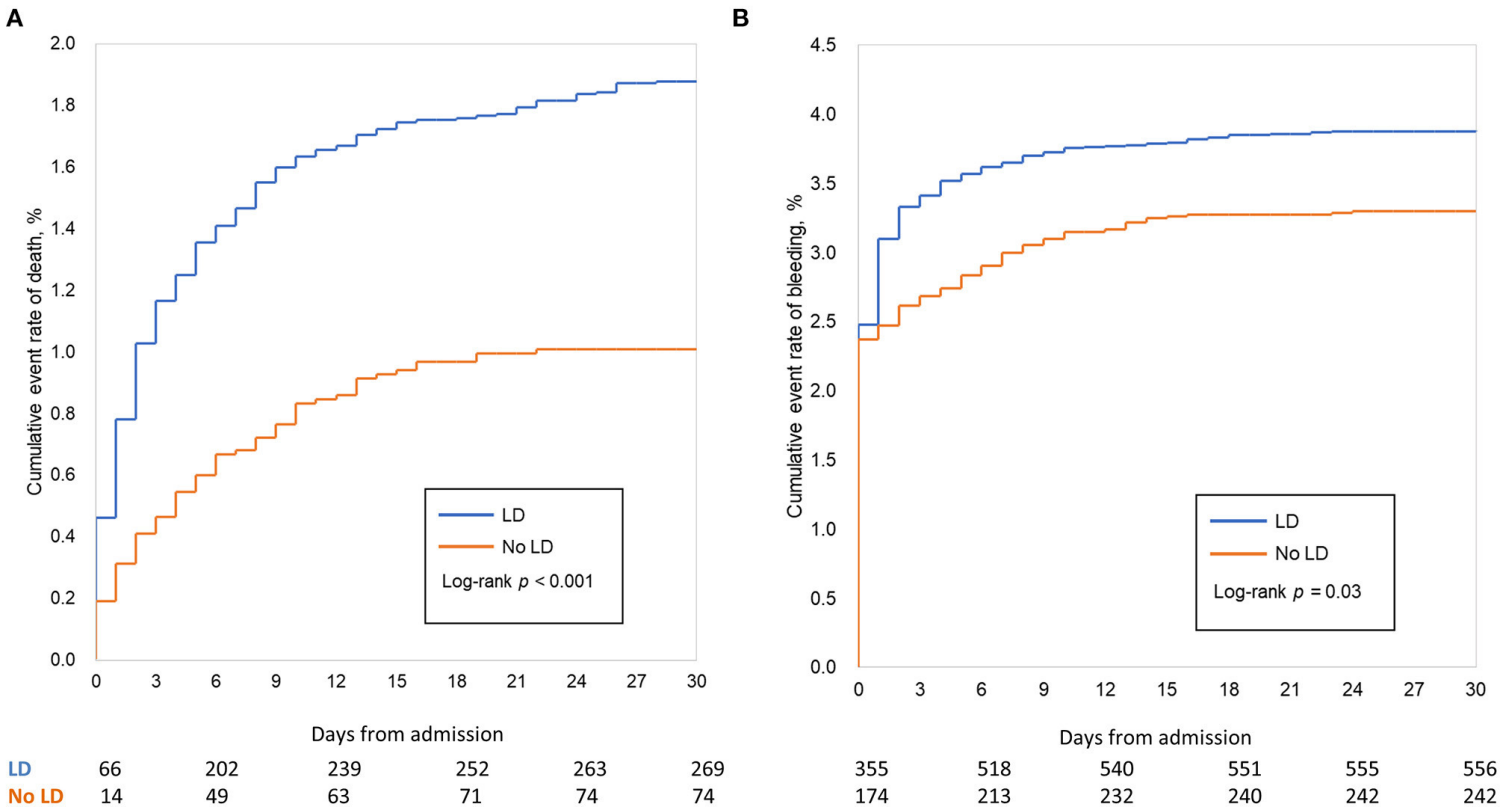
In-hospital clinical outcomes (unadjusted analysis). Cumulative Kaplan–Meier curve estimates of effectiveness outcomes during the 15-day in-hospital period in the whole study population: mortality **(A)**, major bleeding **(B)**. In-hospital mortality was significantly lower in patients treated without dual loading dose of antiplatelet therapy (No LD) compared with those who were. The overall incidence of in-hospital bleeding after percutaneous coronary intervention (PCI) was 3.69%; after adjustment for patient characters and hospital effects (Cox model), LD was associated to higher rate of bleeding.

Table 2 and Figure 2 showed the in-hospital outcomes between two groups. Mortality before discharge was higher in patients who were with LD than those without LD (1.89 vs. 1.02%, *p* < 0.001), with an adjusted HR (95% CI) of 1.71 (1.12, 2.42), (Table 2; Figure 2A). In the multivariate-adjusted analysis, LD was not associated with a higher risk of in-hospital ischemic stroke, with an adjusted HR of 2.01 (0.86, 4.69). The estimated risk did not differ between the two groups after adjustment in other in-hospital events, such as the MI (0.25 vs. 0.25%, *P* = 0.93) and stent thrombosis (0.11 vs. 0.05%; *P* = 0.19).

**Table 2:** Incidence of in-hospital clinical outcomes, and risk of LD treatment.

**Outcomes**	**Study group**		***p*** **-value**	**HR (95% CI)**	
	**LD (*n* = 14,322)**	**No LD (*n* = 7,332)**		**Unadjusted**	**Adjusted Cox model with IPTW^a^**
Death	271 (1.89)	75 (1.02)	<0.001	1.86 (1.44, 2.40)	1.71 (1.21, 2.42)
Myocardial infarction	36 (0.25)	18 (0.25)	0.93	1.02 (0.58, 1.80)	0.84 (0.39, 1.80)
Stent thrombosis	16 (0.11)	4 (0.05)	0.19	2.05 (0.68, 6.13)	1.54 (0.36, 6.67)
Ischemic stroke	34 (0.24)	13 (0.18)	0.37	1.34 (0.71, 2.54)	2.01 (0.86, 4.69)
Bleeding	557 (3.89)	242 (3.3)	0.03	1.18 (1.01, 1.37)	1.25 (1.03, 1.53)
Major bleeding	364 (2.54)	182 (2.48)	0.79	1.02 (0.86, 1.22)	1.07 (0.84, 1.36)
Fatal bleeding	23 (0.16)	6 (0.08)	0.13	1.96 (0.80, 4.82)	2.01 (0.63, 6.47)

*Values are numbers (percentages) unless otherwise indicated*.

a *Adjusting gender, medical history, current smoker, cardiogenic shock, Killip class, elevated TnT or TnI, anticoagulant during hospitalization, year of enrolment, and hospital type (secondary or tertiary hospital)*.

Higher rates of bleeding, major bleeding and fatal bleeding were observed in LD group than in the No LD group in the whole study population (3.89 vs. 3.30%, *P* = 0.03; 2.54 vs. 2.48%, *P* = 0.79; 0.16 vs. 0.08%, *P* = 0.13, respectively). After adjusting patients' characters, antithrombotic treatment and hospital effect with cox model, LD was associated with a higher rate of bleeding, with an adjusted HR of 1.25 (1.03, 1.53); (Table 2; Figure 2B). However, the estimated risk did not differ between the two groups either for major bleeding [adjusted HR (95% CI): 1.07 (0.84, 1.36)] or fatal bleeding [adjusted HR (95% CI): 2.01 (0.63, 6.47)] (Table 2). The location and intervention of bleeding were showed in Table 3. Gastrointestinal bleeding, subcutaneous hemorrhage and access site bleeding were reported more in LD group (0.98 vs. 0.67%, *P* = 0.02; 0.51 vs. 0.15%, *P* < 0.001; 0.52 vs. 0.25%, *P* = 0.02, respectively). There was no statistically difference between groups in intracranial or retroperitoneal bleeding, as well as in the surgical intervention and blood transfusion.

**Table 3:** Location and intervention of bleeding.

	**Total (*n* = 21,654)**	**LD** **(*n* = 14,322)**	**No LD (*n* = 7,332)**	***p*** **-value**
Intracranial bleeding	52 (0.24)	40 (0.28)	12 (0.16)	0.1
Gastrointestinal bleeding	189 (0.87)	140 (0.98)	49 (0.67)	0.02
Subcutaneous hemorrhage	84 (0.39)	73 (0.51)	11 (0.15)	<0.001
Access site bleeding/hematoma^#^	41 (0.36)	24 (0.52)	17 (0.25)	0.02
Retroperitoneal bleeding^#^	1 (0.01)	1 (0.02)	0 (0)	0.23
Other bleeding^#^	63 (0.55)	46 (0.99)	17 (0.25)	<0.001
Bleeding requiring surgical intervention	44 (0.2)	28 (0.2)	16 (0.22)	0.72
Blood transfusion	112 (0.52)	69 (0.48)	43 (0.59)	0.31

*Values are numbers (percentages) unless otherwise indicated*.

# *Calculated by 11,368 available data from Nov 2014 to Jul 2017*.

### Subgroup Analysis

**Figure 3:**
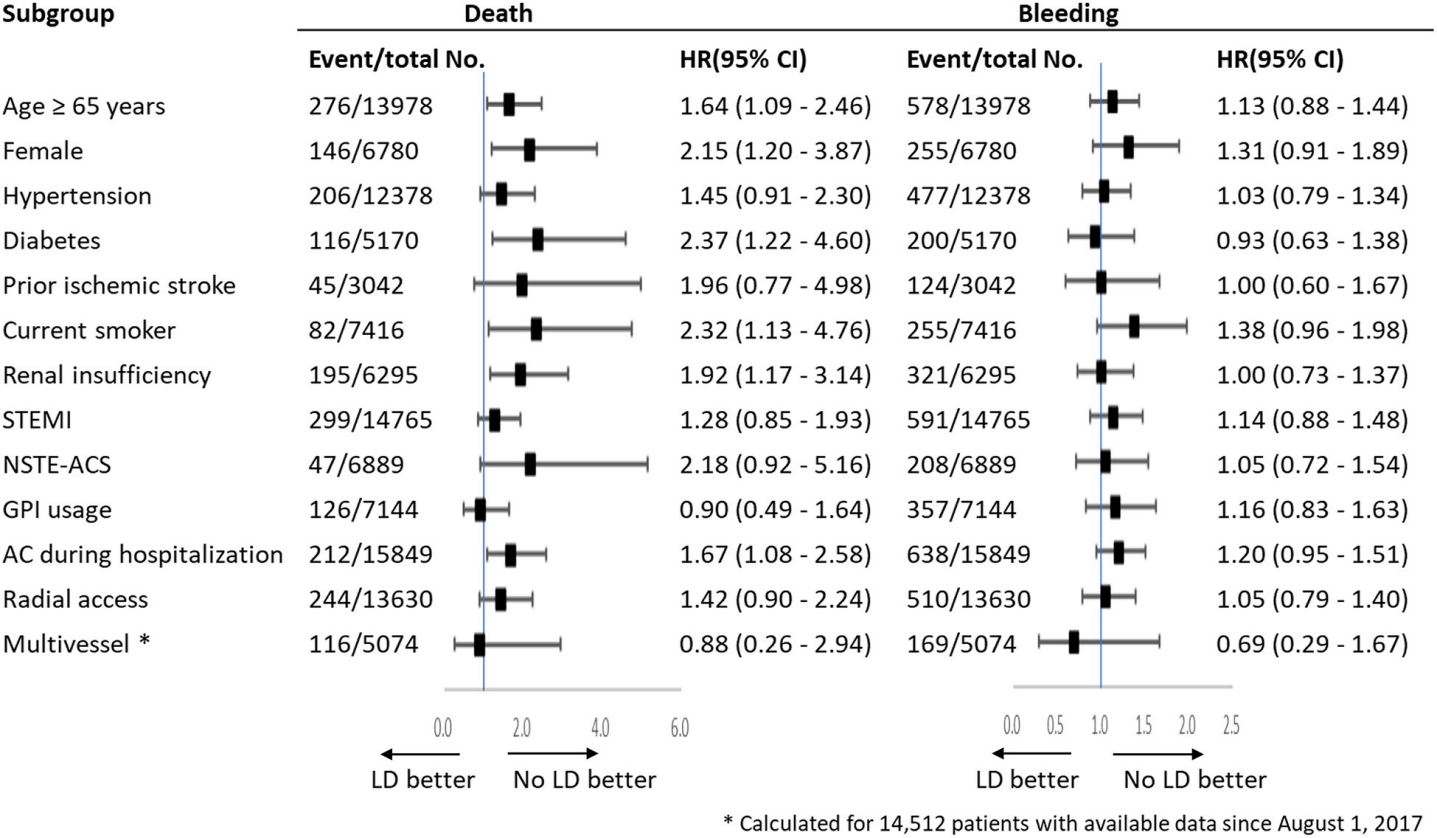
Subgroup analysis.

Subgroup analysis was conducted in thirteen subgroups of the whole study population (Figure 3). The results showed LD was associated with an increased risk of mortality with increasing risk of bleeding, which were consistent with the main results. In these analyses, there were no significant interactions with baseline or procedural variables, except advanced age, female, diabetes, smoking, renal insufficiency and anticoagulation during hospitalization.

### Role of Potent P2Y_12_ Inhibitor

For patients treated with clopidogrel, the rate of mortality was 1.93% for individuals with LD and 1.02% for those without LD, with an adjusted HR (95% CI) of 1.70 (1.18, 2.43). For patients treated with ticagrelor, the rate of the all-cause death was 1.84% for individuals with LD and 1.02% for those without LD, with an adjusted HR (95% CI) of 1.57 (1.00, 2.47) (Table 4). The higher risk of bleeding of LD remained consistent across the P2Y_12_ inhibitor types, especially in ticagrelor [adjusted HR (95% CI): 1.31 (1.02, 1.69)]. The use of LD was not associated with increased risk of MI, stent thrombosis and major bleeding for patients treated with either clopidogrel or ticagrelor.

**Table 4:** Unadjusted and adjusted hazard ratios for in-hospital endpoint in patients with LD vs. no-LD stratified by P2Y_12_ inhibitor type.

**Outcomes**	**Clopidogrel**					**Ticagrelor**				
	**LD (*n* = 8,493)**	**No LD** **(*n* = 7,332)**	***P*** **-value**	**Unadjusted** **HR (95%CI)**	Adjusted HR (95%CI)*	**LD** **(*n* = 5,829)**	**No LD (*n* = 7,332)**	***P*** **-value**	**Unadjusted HR (95%CI)**	**Adjusted** HR (95%CI)*
Death	164 (1.93)	75 (1.02)	<0.001	1.90 (1.44, 2.49)	1.70 (1.18, 2.43)	107 (1.84)	75 (1.02)	<0.001	1.80 (1.34, 2.42)	1.57 (1.00, 2.47)
Myocardial infarction	20 (0.24)	18 (0.25)	0.9	0.96 (0.51, 1.81)	0.73 (0.31, 1.70)	16 (0.27)	18 (0.25)	0.74	1.12 (0.57, 2.19)	1.32 (0.53, 3.26)
Stent thrombosis	9 (0.11)	4 (0.05)	0.26	1.94 (0.60, 6.31)	1.22 (0.24, 6.33)	7 (0.12)	4 (0.05)	0.2	2.20 (0.64, 7.52)	2.43 (0.49, 11.97)
Ischemic stroke	27 (0.32)	13 (0.18)	0.08	1.79 (0.93, 3.48)	1.65 (0.68, 4.02)	7 (0.12)	13 (0.18)	0.4	0.68 (0.27, 1.70)	1.46 (0.44, 4.82)
Bleeding	319 (3.76)	242 (3.3)	0.12	1.14 (0.96, 1.35)	1.17 (0.95, 1.45)	238 (4.08)	242 (3.3)	0.02	1.24 (1.04, 1.48)	1.31 (1.02, 1.69)
Major bleeding	206 (2.43)	182 (2.48)	0.82	0.98 (0.80, 1.19)	1.01 (0.78, 1.31)	158 (2.71)	182 (2.48)	0.41	1.09 (0.88, 1.35)	1.02 (0.75, 1.39)
Fatal bleeding	15 (0.18)	6 (0.08)	0.1	2.16 (0.84, 5.56)	2.32 (0.72, 7.50)	8 (0.14)	6 (0.08)	0.33	1.68 (0.58, 4.83)	0.99 (0.20, 4.78)

*Values are numbers (percentages) unless otherwise indicated*.

* *Adjusting gender, medical history, current smoker, cardiogenic shock, Killip class, elevated TnT or TnI, anticoagulant during hospitalization, year of enrolment, and hospital type (secondary or tertiary hospital)*.

## Discussion

Among 21,654 HBR patients undergoing PCI in the CCC-ACS registry analyzed in this study, LD was associated with an increased mortality but not with other ischemic event including in-hospital MI, stent thrombosis or ischemic stroke. Bleeding complications were more observed in the LD group than no LD group, especially with a location of gastrointestinal, subcutaneous and access site.

Optimal antiplatelet therapy before PCI is predominantly focused on reducing early ischemic complications such as reinfarction, stent thrombosis, and stroke. The administration of dual loading antiplatelet therapy has demonstrated efficacy in terms of decreased ischemic events in ACS with a paradoxical increase in bleeding (8). Whereas, the ACC/AHA guidelines recommended of LD treatment in ACS, the ESC guidelines current during the study period stated a preference for no routine pre-treatment with a P2Y_12_ receptor inhibitor in NSTE-ACS patients in whom coronary anatomy is not known and an early invasive management is planned (Class III, Level of evidence A) (1, 2). The randomized Comparison of Prasugrel at the Time of Percutaneous Coronary Intervention or as Pretreatment at the Time of Diagnosis in Patients with Non-ST Elevation Myocardial Infarction (ACCOAST) trial showed no apparent benefit of ischemic prevention for pre-treatment in NSTE-ACS, but a substantially higher bleeding risk with prasugrel pre-treatment (9). With respect to pre-treatment data for ticagrelor, prasugrel, and clopidogrel reported from the Swedish Coronary Angiography and Angioplasty Registry (SCAAR) of 64,857 NSTE-ACS and 44,804 STEMI patients (10, 11). In line with the randomized trials, this observational data reported that P2Y_12_ inhibitor pre-treatment in ACS patients was not associated with improved clinical outcomes. Data from the Downstream vs. Upstream Strategy for the Administration of P2Y_12_ Receptor Blockers In Non-ST Elevated Acute Coronary Syndromes With Initial Invasive Indication (DUBIUS) study showed minimal numeric difference of event rates between two treatment groups. These findings led to premature termination and suggested the unlikelihood of enhanced efficacy of one strategy over the other (12). In our study, not surprisingly, there was an observed increase in mortality and bleeding risk relating to the use of LD, representing local medical routine as antiplatelet pre-treatment in full- dose at admission in both NSTE-ACS and STEMI. Our data should lead to greater awareness of the prognostic importance of bleeding complications in ACS and HBR. Dedicated randomized trials with contemporary ACS management are needed to confirm these findings in this population.

Multiple differences exist between East Asian and Western population, genetic variation especially with respect to their thrombogenicity and hemorrhagic diathesis (13, 14). In Korean, standard-dose ticagrelor as compared with clopidogrel was associated with a higher risk of clinically significant bleeding in ACS patients with early invasive management. Of note, the numerically higher rate of ischemic events driving underpowered conclusion regarding efficacy. In addition, data from Japanese population proved that prasugrel at reduced-dose was comparable with standard-dose clopidogrel in ACS undergoing PCI (15). Furthermore, Japanese regional registry reported 43% of patents present as HBR, and with 3-fold risk of bleeding event than the non-HBR patients (5). On the other hand, high platelet reactivity was observed in 37.7% patient in the prospective, multicenter registry of Japanese patients with PCI, and has been proven related to reverse poor prognosis in patients with and without ACS (16). LD might be useful in preventing thrombotic cardiovascular events but worse net clinical benefit owing to a high frequency of bleeding complications. In the population of East Asian, this possibility is supported by the present analysis showing the worse outcome of LD in HBR with ACS undergoing PCI. Personalized stratification of risk schemes for ischemic and bleeding is needed in ACS, especially among East Asian population, who shares nearly 20% population worldwide.

Early identification of HBR patients are key elements of bleeding complication prevention (17). In our study, advanced age, female, diabetes, smoking, renal insufficiency, anticoagulation during hospitalization and ticagrelor-related bleeding may contribute to the worse outcome in the LD group. Patient's demographic and comorbidities, such as female and diabetes, are proven factors for bleeding risk classification and easy are noticed by medical staff at admission (18, 19). Antithrombotic therapies, especially at admission, is usually with full-dose owing the high risk of thrombosis and neglecting their contribution of bleeding complications. With the use of more effective and timely reperfusion therapies in ACS, post-discharge bleeding has an equivalent prognostic impact as post-discharge myocardial infarction on mortality in patients with or without PCI (20). Furthermore, compared with a safety strategy of clopidogrel reloading for patients with acute myocardial infarction already on clopidogrel therapy, ticagrelor showed higher platelet inhibition within the first 24 h after ticagrelor reloading (21, 22). Bleeding risk stratification is warrant ahead of LD decisions to reduce bleeding and thereby improve clinical outcomes. Understanding these differences in antithrombotic strategies including LD and their impacts on clinical outcomes will aid in selection of the optimal tailored antithrombotic therapy for HBR patients with ACS.

### Study Limitations

Several limitations of this observational study should be noted. First, selection bias and residual confounding exist in our analyses as all observational studies. Nevertheless, this study provides real-world data from a large cohort with complete coverage of ACS patients in China. Second, we did not adjust for the switch in P2Y_12_ inhibitors, which is happened frequently among patients treated with PCI. However, chronic, or incorrect use of antiplatelet drugs, such as loading with both ticagrelor and clopidogrel, were excluded in order to minimize their confounding effect. The CCC-ACS does not gather all information for ARC-HBR classification, and we used modified criteria instead. Some factors related to the patient's adjunctive treatment regimens changed throughout the study period. We accounted these changes as a covariate in the statistical models.

## Conclusion

In conclusion, among HBR patients undergoing PCI for ACS in CCC-ACS registry between 2014 and 2019, LD was associated with an increased risk of in-hospital mortality and bleeding complications. Bleeding risk stratification is warrant ahead of LD decisions in daily practice. Further randomized studies to assess the safety and efficacy of full loading in HBR patients would probably add to the existing evidence for personalized antiplatelet therapy.

## Data Availability Statement

The data analytic methods and study materials will be made available for onsite audits by third parties for the purposes of reproducing the results or replicating the procedure.

## Ethics Statement

The study was approved by the Institutional Review Board at Beijing Anzhen Hospital, Capital Medical University. Written informed consent for participation was not required for this study in accordance with the national legislation and the institutional requirements.

## Author Contributions

YY, GW, and SN contributed to the manuscript draft and analysis. YY, WG, XH, SL, GW, YM, and SN contributed to the manuscript development. YH and JL were responsible for overseeing and monitoring the implementation of the CCC-ACS registry. All authors gave final approval and agree to be accountable for all aspects of the work ensuring integrity and accuracy.

## Funding

The CCC-ACS project is a collaborative study of the AHA and CSC. The AHA has been funded by Pfizer and AstraZeneca for quality improvement initiatives through an independent grant. YY was funded by grants from the National Natural Science Foundation of China (82100260), Beijing Hospitals Authority Youth Program (QML20210605) and Beijing Municipal Administration of Hospitals Incubating Program (PZ2019005). WG was funded by grants from the National Natural Science Foundation of China (81970292, 81600213), Beijing Hospitals Authority Youth Program (QML20190603), Optimizing Antithrombotic Research Fund (BJUHFCSOARF201901-08). SN was funded by National Natural Science Foundation of China (81670222), Beijing Municipal Administration of Hospitals Clinical Medicine Development of Special Funding Support (ZYLX201710), Beijing Municipal Administration of Hospitals' Ascent Plan (DFL20180601), Natural Science Foundation of Beijing, China (7191002), and the Capital Health Research and Development of Special Fund (2018-1-2061). The remaining authors declare that the research was conducted in the absence of any commercial or financial relationships that could be construed as a potential conflict of interest.

## Conflict of Interest

The authors declare that the research was conducted in the absence of any commercial or financial relationships that could be construed as a potential conflict of interest.

## Publisher's Note

All claims expressed in this article are solely those of the authors and do not necessarily represent those of their affiliated organizations, or those of the publisher, the editors and the reviewers. Any product that may be evaluated in this article, or claim that may be made by its manufacturer, is not guaranteed or endorsed by the publisher.
