# Supplementary material for: Dual Loading Antiplatelet Therapy in Patients With Acute Coronary Syndrome and High Bleeding Risk Undergoing Percutaneous Coronary Intervention: Findings From the Improving Care for Cardiovascular Disease in China Project
Source: Front Cardiovasc Med. 2022 Mar 23;9:774123. doi: 10.3389/fcvm.2022.774123 (PMC8984244; doi:10.3389/fcvm.2022.774123)
Supplement: Supplementary file 1 [file Table_1.DOCX]

**Supplementary Table 1. Comparison of definitions between the ARC-HBR criteria and the current study.**

| **ARC-HBR criteria** | **Present study** | **Category** | **Comments** |
| --- | --- | --- | --- |
| Age ≥75 y | Age ≥75 y | Minor | Identical |
| Moderate CKD (eGFR 30–59 mL/min) | eGFR 30–59 mL/min | Minor | Identical |
| Hemoglobin 11–12.9 g/dL for men and 11–11.9 g/dL for women | Hemoglobin 11–12.9 g/dL for men and 11–11.9 g/dL for women | Minor | Identical |
| Spontaneous bleeding requiring hospitalization or transfusion within the past 12 mo not meeting the major criterion | Bleeding tendencies before admission | Minor | Modified |
| Long-term use of oral NSAIDs or steroids | Long-term use of aspirin before admission | Minor | Modified |
| Any ischemic stroke at any time not meeting the major criterion | Prior ischemic stroke or TIA | Minor | Modified |
| Anticipated use of long-term oral anticoagulation | Warfarin usage before admission | Major | Modified |
| Severe or end-stage CKD (eGFR <30 mL/min) | eGFR <30 mL/min | Major | Identical |
| Hemoglobin <11 g/dL | Hemoglobin <11 g/dL | Major | Identical |
| Spontaneous bleeding requiring hospitalization or transfusion in the past 6 mo or at any time, if recurrent | N/A | Major | N/A |
| Moderate or severe baseline thrombocytopenia (platelet count <100×10^9^/L) | Platelet count <100×10^9^/L | Major | Identical |
| Chronic bleeding diathesis | N/A | Major | N/A |
| Liver cirrhosis with portal hypertension | N/A | Major | N/A |
| Active malignancy (excluding nonmelanoma skin cancer) within the past 12 mo | N/A | Major | N/A |
| Previous spontaneous ICH (at any time)/Previous traumatic ICH within the past 12 mo/Presence of a brain arteriovenous malformation/Moderate or severe ischemic stroke within the past 6 mo | Prior hemorrhagic or ischemic stroke | Major | Modified |
| Nondeferrable major surgery on DAPT | N/A | Major | N/A |
| Recent major surgery or major trauma within 30 d before PCI | Medical history of surgery or tooth extraction | Major | Modified |

CKD: chronic kidney disease; N/A: Not available; eGFR: estimated glomerular filtration rate; NSAIDs : Nonsteroidal anti-inflammatory drugs; TIA: transient ischemic attack; ICH: intracranial haemorrhage; DAPT: dual antiplatelet therapy; PCI: percutaneous coronary intervention
